# Chinese Milk Vetch as Green Manure Mitigates Nitrous Oxide Emission from Monocropped Rice System in South China

**DOI:** 10.1371/journal.pone.0168134

**Published:** 2016-12-13

**Authors:** Zhijian Xie, Farooq Shah, Shuxin Tu, Changxu Xu, Weidong Cao

**Affiliations:** 1 Institute of Soil & Fertilizer and Resources & Environment, National Engineering and Technology Research Center for Red Soil Improvement, Jiangxi Academy of Agricultural Sciences, Nanchang, Jiangxi, P.R. China; 2 College of Resources and Environment, Microelement Research Center, Huazhong Agricultural University, Wuhan, Hubei, P.R. China; 3 Department of Agriculture, Abdul Wali Khan University Mardan, Khyber Pakhtunkhwa, Pakistan; 4 Hubei Collaborative Innovation Center for Grain Industry, Jingzhou, Hubei, P.R. China; 5 Institute of Agricultural Resources and Regional Planning, CAAS, Beijing, P.R. China; University of Delhi, INDIA

## Abstract

Monocropped rice system is an important intensive cropping system for food security in China. Green manure (GM) as an alternative to fertilizer N (FN) is useful for improving soil quality. However, few studies have examined the effect of Chinese milk vetch (CMV) as GM on nitrous oxide (N_2_O) emission from monocropped rice field in south China. Therefore, a pot-culture experiment with four treatments (control, no FN and CMV; CMV as GM alone, M; fertilizer N alone, FN; integrating fertilizer N with CMV, NM) was performed to investigate the effect of incorporating CMV as GM on N_2_O emission using a closed chamber-gas chromatography (GC) technique during the rice growing periods. Under the same N rate, incorporating CMV as GM (the treatments of M and NM) mitigated N_2_O emission during the growing periods of rice plant, reduced the NO_3_^-^ content and activities of nitrate and nitrite reductase as well as the population of nitrifying bacteria in top soil at maturity stage of rice plant versus FN pots. The global warming potential (GWP) and greenhouse gas intensity (GHGI) of N_2_O from monocropped rice field was ranked as M<NM<FN. However, the treatment of NM increased rice grain yield and soil NH_4_^+^ content, which were dramatically decreased in the M pots, over the treatment of FN. Hence, it can be concluded that integrating FN with CMV as GM is a feasible tactic for food security and N_2_O mitigation in the monocropped rice based system.

## Introduction

Mitigating climate change and ensuring food security are the two widely acknowledged key challenges of the 21^st^ century. Cereals (e.g. rice, wheat and corn) are by far the world’s most important food sources, contributing on average 50% of daily energy intake in general and up to 70% in some developing countries [1]. As the population rises, China will need to produce ~26.9% more rice by 2030 (compared with 2000) to meet the domestic need and ensure food security, if rice consumption per capita stays at the current level [2]. Increasing the use of fertilizer N (FN) in rice production is essential in increasing rice yield, partly due to the limited cultivated area of rice paddies [3]. Since the early 1980s, Chinese agriculture has intensified greatly within a limited land area due to large inputs of chemical fertilizer [4]. However, large input of FN and low nitrogen use efficiency (NUE) are causing serious environmental problems [5], include soil and water pollution, loss of biodiversity and greenhouse gases (GHGs) emissions [6].

Nitrous oxide (N_2_O), a naturally occurring and chemically stable GHG which has a lifetime of more than 100 yr in the atmosphere, plays a significant role in terms of global warming [7]. N_2_O is almost 298 times more potent in radiative forcing than carbon dioxide (CO_2_) over a 100-year time horizon [8]. Furthermore, N_2_O participates in many photochemical reactions in the atmosphere and is considered to be the greatest anthropogenic factor influencing the depletion of stratospheric ozone in 21^st^ century [9]. Human activities are probably among the key causes of the increase in the atmospheric N_2_O concentration. Agricultural soils have been identified as the major anthropogenic sources of N_2_O entering atmosphere, which contribute to approximately 6%-11% of global total N_2_O emissions [10]. Furthermore, N fertilization contributed about 36% of the direct N_2_O emissions from worldwide agricultural soils and the higher FN input increased N_2_O emission in the double-rice system [11–12]. The annual direct and indirect N_2_O emissions from inorganic fertilizer applied at the rate of 79.1×10^3^ kg N and of manure spreading at 33.3×10^3^ kg N in global arable systems equate to 4×10^3^ kg N_2_O-N [13]. Thus, there is a worldwide concern over N_2_O emissions from agricultural soils, especially in China where the applied inorganic FN applied accounts for 30% of the total global use [14].

N_2_O emissions come from microbially mediated soil processes, particularly via denitrification and nitrification [15]. The flux of N_2_O between the soil and the atmosphere largely depends on soil water content, O_2_ availability, N substrate availability nitrate (NO_3_^-^) and ammonium (NH_4_^+^), and organic C substrate availability [16]. The application of mineral N increases substrate availability for nitrification and denitirification [17], and thus provides more available N for soil microbes, and leads to higher N_2_O efflux [18]. Moreover, the ammonia oxidizing bacteria (AOB) and archaea (AOA) produce N_2_O as a byproduct through the oxidation of ammonia to nitrite via the nitrification process [19], whereas the high oxygen (O_2_) demand during decomposition of organic manure or GM decrease the populations of AOB and denitrifying substrates (NO_3_^-^) in paddy soils [20], which might result in a decrease of the N_2_O emission rate in rice-duck farming fields [21]. Furthermore, the recycling of N in animal manure, human excreta and compost to reduce inorganic fertilizer decreases N_2_O emissions from agricultural ecosystems [13]. The increasing soil organic carbon (SOC) by compost application, then partially increases N supply to crops instead of adding inorganic FN, making it an effective measure to mitigate N_2_O emissions from arable soils in the winter wheat-summer maize cropping system in north China plain [22].

Furthermore, crop residue management can greatly regulate N_2_O emission [23]. Tang et al. (2015) [24] reported that incorporating winter cover crop residues (e.g. rape, potato, etc.) increased the total N_2_O emission from double-rice cropping system in southern China, however, incorporating leguminous milk vetch residue to paddy soil inhibited the N_2_O emission [25] and legumes reduced the overall N_2_O emission intensity in subtropical cereal cropping system [26]. Consequently, the non-consistent results implied that the effect of crop residue retention on N_2_O emission maybe dependent on the local conditions (e.g. soil conditions, cropping ecosystems, crop types, N management, etc.) [27].

Growing winter crop in the fallow seasons after rice harvest and recycling them into soil as green manure (GM) before rice transplanting next year are traditional practices as well as rice straw retention. Many experiments were conducted to study the effects of winter crops as GM on soil biological properties, greenhouse gas (CH_4_) emission and crop productivity [28–29]. Leguminous winter crop, such as Chinese milk vetch (CMV, *Astragalus sinicus* L.), is a common winter crop in paddy fields in south of China. In fact, using leguminous winter crop (e.g. CMV) residue as GM has been considered a beneficial practice to reduce the requirement for synthesized FN, improve soil N-supplying capacity and support sustainable rice productivity in the double-rice cropping system in southern China [30]. Hence, the CMV as winter crop in paddy fields has the potential to reduce the rice production costs as well as the environmental costs in rice-based cropping system. However, few studies have estimated N_2_O emission of CMV residue retention as GM on soil as well as its effect on rice grain yield and soil properties of succeeding monocropped rice system.

Monocropped rice system is one of the most important agro-ecosystems, which markedly depends on more nitrogen (N) supply to obtain high-yield of rice production in the red soil regions of south China [31]. However, the N use efficiency of the rice cropping system is just about 30% in China [32], and the part of the applied N that is not taken up by the crop or immobilized in soil organic N pools is vulnerable to boosting N emission (e.g. N_2_O, etc.) to the atmosphere [33]. Thus how to achieve a multifaceted goal of ensuring greater rice yields and ecological benefits of FN application as well as reducing N losses and the related environmental risks is imperative for China. As mentioned above, using GM as an alternative to FN may be expected to simultaneously reduce the rice production costs as well as the environmental perils in rice-based cropping system. As it was a preliminary trial and was not possible to consider all the related concerns, we deliberately limited the scope of this study by a short-term pot trial. Therefore, the aims of this study were (1) to quantify N_2_O emissions from monocropped rice system influenced by CMV as GM incorporation in south China, (2) to evaluate the effect of CMV as GM on the global warming potential (GWP) and greenhouse gas intensity (GHGI) of N_2_O in a paddy field, and (3) to determine the effect of incorporating CMV as GM into soil on some of soil chemical and biological properties of the succeeding monocropped rice soil. The results will contribute to frame mitigation strategies for environmental risks related to N losses (e.g. NO_3_^-^ leaching and runoff, N_2_O emission, etc) and for the sustainable intensification of monocropped rice system.

## Materials and Methods

### Experimental site and climate

**Fig 1:**
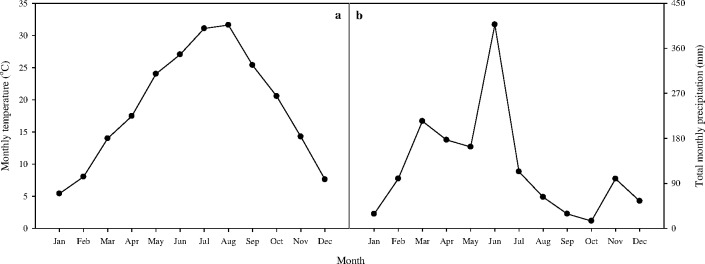
The mean monthly temperature (a) and total monthly precipitation (b) at the experimental site.

The experimental site has a subtropical monsoon climate, characterized by heavy rain from March to June and drought from September to December. The average annual temperature is 17.8°C and the average annual rainfall is 1545.9 mm. The annual sunshine is 1603.4 h, with an average frost-free period lasting 276 d. The average monthly temperature (°C) and total monthly precipitation (mm) at the experimental site from May to September are shown in Fig 1. The greenhouse has an automatic air exhausting system which is capable of maintaining the same temperature as outside and the rice plants were grown under paddy field conditions (e.g. saturation with 3–5 cm of standing water).

### Experimental soil and crop

A bulk paddy soil (0–20 cm), derived from Quaternary red soil was collected from Fengcheng, Jiangxi (N 28°07′, E 115°56′, altitude 25.4 m). The soil samples were air-dried for two weeks and sieved through a compost sieving machine (< 5 mm) and stored air-dry until preparations for the experiment. Soil pH (soil: water ratio was 1: 2.5), total N (Kjeldahl method), extractable P (the Olsen′s method), available K (with ammonium acetate) were determined following procedures described by Page et al. (1982) [34]. The organic carbon content was determined according to the Walkley and Black (1934) method [35]. The soil bulk density (SBD) was measured by taking soil cores with a predefined steel cylinder (height 5 cm, ∅ 7.14 cm) from the undisturbed top soil, oven-dried at 105°C for 24 h and weighed, then calculated as the ratio of dry soil weight (g) to the volume of soil contained in a predefined steel cylinder [36]. All of these parameters were determined before the experiment was carried out in 2013. The main properties of the top soil (0–20 cm) were as follows: pH 5.41, total N 1.35 g kg^-1^, organic carbon 16.0 g kg^-1^, extractable P 8.52 mg kg^-1^, available K 68.0 mg kg^-1^ and the SBD was 1.47 g cm^-3^.

The model green manure (GM) crop used in this trial was CMV (*Astragalus sinicus* L., provided by germplasm section of Institute of Soil & Fertilizer and Resources & Environment) which contained N 3.35 g kg^-1^ and was having a moisture content of 91.6%.

### Experimental design and crop management

A pot experiment was conducted in the greenhouse of Institute of Soil & Fertilizer and Resources & Environment, Jiangxi Academy of Agricultural Sciences in Jiangxi, China (N 28°33′44.47′′, E 115°56′15.09′′) from May to September 2013. The size of stainless steel container was 40 cm × 40 cm × 30 cm which was filled with 47 kg of the air-dried soil (prepared as described above).

The experiment included four treatments arranged in a randomized complete block design with three replications: (1) control (no fertilizer N and CMV), (2) CMV as GM alone (M), (3) fertilizer N alone (FN), (4) integrating fertilizer N with CMV (NM).

The applying rate of fertilizers to monocropped rice was: 356.9 kg N ha^-1^ (urea 46.4% N), 153 kg P_2_O_5_ ha^-1^ (super phosphate 12.0% P_2_O_5_) and 306 kg K_2_O ha^-1^ (potassium chloride 60% K_2_O). All of the chemical N, P and K fertilizers were applied as basal fertilizer one day before transplanting the rice seedlings. The fresh vetch straw was applied at the rate of 105.2 and 22.5 Mg ha^-1^ in the M and NM treatments for the following monocropped rice season respectively, 30 days prior to rice transplanting, mixed mechanically within 20 cm depth of surface paddy soil, and then flooded up to 3–5 cm depth. All treatments (except the control treatment) were fertilized with the same rate of N nutrient which was either from FN/GM alone or both from FN and GM.

The 25-days old rice seedlings (*Oryza sativa*, provided by Zhejiang Nongke Seed Industry Co. Ltd.) were transplanted to the prepared stainless steel containers with six caves (two rice seedlings per cave) for each container on 20^th^ May, 2013. During the growing period of rice plants, the soil water layer was maintained at 3–5 cm depth by monitoring every day and the soil water dried naturally 20 days before harvesting. All rice plants were harvested during the last-ten days of September.

### N_2_O sampling and measurement

The N_2_O emitted from the paddy soil-plant system was collected via the static chamber and gas chromatography method at 9:00–11:00 am during the rice growing season. The chamber (40 × 40 ×100 cm) was made of 5 mm PVC board with a PVC base. The base has a groove in the collar, in which the chamber could be settled. The chamber base was fixed around the container (as mentioned above) and was flushed with the container collar. The chamber was settled into the groove of the collar with water to prevent gas exchange. The chamber contained a small fan for stirring air, a thermometer sensor, and a trinal-venthole. From the second day after transplanting of rice seedlings, gases were sampled weekly, and the time delay between the basal fertilization, alternation of wetting and drying process and the gases sample collection were 4 days. Before sampling, the fan in the chamber was turned on to evenly mix the air before extracting the air with a 25 ml injector at 0, 10, 20, and 30 min after chamber placement over the rice planted pots and closing the chamber. The gas samples were transferred into 20 ml sealed vacuum flask by rotating trinal venthole.

The gas samples were analyzed within 48 h for N_2_O concentrations using a gas chromatograph (Agilent 7890A) equipped with an electron capture detector (ECD) and a back-flush controlled by a 10-port valve. The carrier gas was 95% argon-5% methane at a flow rate of 30 ml min^-1^. The oven temperature was 85°C, and the detector temperature was 320°C. The N_2_O fluxes and cumulative emissions of N_2_O were calculated according to Eqs 1 and 2 respectively:

$$F=\rho \times \frac{V}{S}\times \frac{\Delta c}{\Delta t}\times \frac{273}{273+T}$$ (1)

where *F* stands for N_2_O flux in μg N_2_O-N m^-2^ h^-1^; *ρ* is the density of N_2_O under a standardized state (1.978 g cm^-3^); *V* is the volume of the chamber (m^3^); *S* is the area from which N_2_O was emitted into the chamber (m^2^); $\frac{\Delta c}{\Delta t}$ is the rate of accumulation in ppbv N_2_O-N h^-1^; *T* is the chamber temperature expressed in °C. All average N_2_O fluxes are expressed as mean values of their individual measurement data weighed by the measurement intervals.

$$N_{f}=\frac{1}{2}\times 24\times 10^{-5}\times \sum_{i=1}^{n}[(F_{i}+F_{i-1})\times (d_{i}-d_{i-1})]$$ (2)

where *N*_*f*_ is the cumulative emissions of N_2_O in kg N_2_O-N ha^-1^, *t* is the various sampling times, *d* is the date of sampling. The cumulative N_2_O emissions were calculated from the emissions between every two adjacent intervals of the measurements.

Emission factor were corrected for background emissions using Eq 3:

$$E_{f}(\%)=\frac{\sum {\left(N_{2}O\right)}_{N}-\sum {\left(N_{2}O\right)}_{C}}{N}\times 100$$ (3)

where *E*_*f*_ is the N_2_O emission factor, ∑(*N*_2_*O*)_*N*_ and ∑(*N*_2_*O*)_*C*_ are the cumulative N_2_O emissions from the pots with or without FN and CMV respectively, *N* is the amounts of nitrogen nutrient applied to per unit area (kg ha^-1^) which was either from FN/GM alone or from both GM and FN.

The greenhouse potential (GWP, kg CO_2_-eq ha^-1^) and greenhouse gas intensity (GHGI, kg CO_2_-eq kg^-1^ grain yield) of N_2_O in the rice based cropping system were calculated according to Eqs 4 and 5 respectively:

$$GWP=298\times N_{2}O$$ (4)

where, the number 298 represent the IPCC factor for the conversion of N_2_O to CO_2_ equivalent [37].

$$GHGI=\frac{GWP}{Yield}$$ (5)

### Sampling procedure and detection

Soil samples (0–20 cm) from all pots were collected for an analysis of soil mineral N (ammonium and nitrate) content at the maturity stage of rice plants with a soil corer (∅ 3 cm). At each sampling pot, five soil cores were randomly collected and then mixed thoroughly inside a plastic bucket to form individual bulked pot soil samples. After removing visible roots and stones, the soil samples were separated into two parts. One of them was air-dried at room temperature and then sieved < 0.149 mm prior to chemical analysis, and the other part was refrigerated at 4°C.

The soil samples were extracted by 2 mol·L^-1^ KCl. An ultraviolet spectrophotometer (Shimadzu UV mini-1240, with ±0.005 absorbance of photometric accuracy) was used to measure the concentrations of NH_4_^+^ and NO_3_^-^. The NO_3_^-^ concentration was directly determined by the difference in absorbance at 220 nm and 275 nm wavelengths [38]. The NH_4_^+^ concentration was determined by an indo-phenol-blue colorimetric method at 625 nm wavelength [39]. The procedure detection limits for both NH_4_^+^ and NO_3_^-^ were lower than 0.05 mg N L^-1^, with a relative error of 1%. The activity of nitrate reductase (*NaR*) was analyzed by the procedures as described by Abdelmagid and Tabatabai (1987) [40]. In briefly, soil sample (5 g) in a 250 ml French square bottle was treated with 2 ml of absolute ethanol containing the appropriate amount of 2,4-Dinitrophenol (DNP, Eastman Kodak Co., Rochester, NY) and 10 ml of 5 mM KNO_3_. The bottle was swirled for a few seconds to mix the contents and shaken horizontally in a reciprocal shaker for 30 min. Then the soil suspension was filtered by using Whatman filter paper. The NO_2_^-^-N in the resulting soil filtrate was determined by transferring 1 ml of the filtrate into a 50 ml volumetric flask and determining the NO_2_^-^-N by the colorimetric method of Friess-Illosvay, as modified by Barnes and Folkard (1951) [41], by using a Klett-Summerson colorimeter fitted with a green filter.

The activity of nitrite reductase (*NiR*) was analyzed by the procedures as described by Tabatabai (1982) [42]. Briefly, soil sample (1 g) in a 100 ml pressure reducing triangle bottle was treated with 20 mg CaCO_3_ and 1 ml 0.5% NaNO_2_. The bottle was shaken for a few seconds and added 1 ml 1% glucose solution, then vacuumed for 3 min and held in the incubator at 30°C for 24 h. At the end of the culture, 50 ml of deionized water and 1 ml of saturated solution containing potassium, aluminum and vanadium were added and shaken up. The soil suspension was filtered by using Whatman filter paper. Then the NO_2_^-^-N in the resulting soil filtrate was determined by transferring 1 ml of the filtrate as well as 5 ml deionized water and 4 ml Griess reagent (contained 0.2% naphthylethylenediamine dihydrochloride and 2% sulphanilamide in 5% phosphoric acid) into a 50 ml volumetric flask and homogenizing, then determining the NO_2_^-^-N by the colorimetric method at 550–600 nm wavelengths.

The populations of cultivable nitrifying and denitrifying bacteria in fresh soil were determined using the Most Probable Number technique as described by Alexander (1982) [43]. Briefly, soil samples (5 equivalent dry mass) were ground and homogenized in 25 ml of NaCl (0.8%) for 1.5 min with a Waring blender (Eberbach Corporation). Soil suspensions were then serially diluted 5-fold. The number of denitrifying bacteria was determined after incubation into 12 × 8-well microtitre plates which were containing 28 μg N ml^-1^ of KNO_3_. For each dilution, 8 wells were inoculated (100 μl inoculum). The plates were incubated in anaerobic conditions for denitrifying bacteria (BBL GasPak Pouch, Anaerobic System, Becton Dickinson, USA) at 28°C in the dark for 13 days. The presence of denitrifying bacteria were revealed using Morgan′s reagent [44]. The population of nitrifying bacteria was determined after incubation into 4 × 6-well microtitre plates containing 500 μl of the double strength mineral salts selective medium [45], with (NH_4_)_2_SO_4_ (106 μg ml^-1^ NH_4_^+^-N) or NaNO_2_ (70 μg ml^-1^ NO_2_^-^-N). For each dilution, 6 wells were inoculated (500 μl inoculum). The plates were incubated at 28°C in the dark for 9 weeks. The presence of nitrifying bacteria was revealed by the presence of NO_2_^-^ using Morgan′s reagent and Griess-Ilosvay′s reagent [44, 46]. The most probable number of denitrifying and nitrifying bacteria was estimated by Cochran′s method [47].

At physiological maturity, grains were separated from straw in the whole pot. The grains were sun-dried and weighed separately to obtain the yield, expressed as Mg ha^-1^.

### Statistical analysis

All data represented herein were arithmetic means ± standard deviation (SD) of three replicates. Data were statistically analyzed as a randomized complete block by using the one-way PROC ANOVA procedure of SAS statistical package (Version 9.1, Cary, USA). Mean values were distinguished by using the Tukey-HSD test at *P*<0.05 levels. Sigmaplot 10.0 and MS excel were used to generate the graphs and tables respectively.

## Results

### N_2_O emission

**Fig 2:**
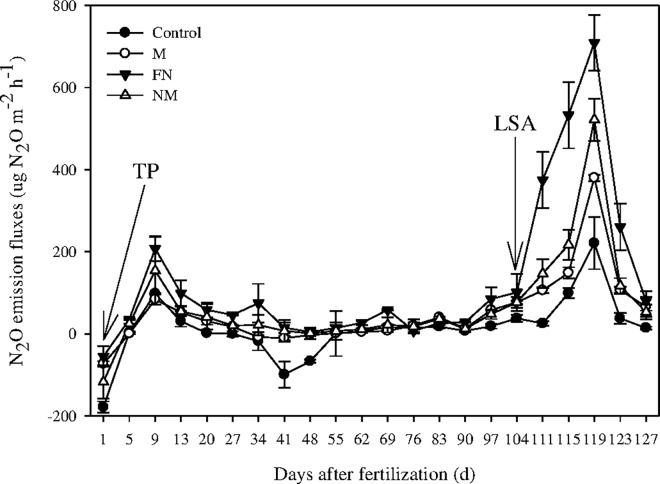
The N_2_O emission fluxes during the growing period of rice plants. TP was the time of transplanting rice seedlings, and LSA was the time of 20 days before rice harvest. Bars indicate standard error (n = 3).

Fig 2 shows that the first peaks of N_2_O emission, were 98.2 μg N_2_O-N·m^-2^·h^-1^, 112.2 μg N_2_O-N·m^-2^·h^-1^, 207.7 μg N_2_O-N·m^-2^·h^-1^ and 153.9 μg N_2_O-N·m^-2^·h^-1^ in the control, M, FN and NM pots respectively from paddy soil, which were observed 9 days after transplanting rice seedlings, and then declined sharply and remained at a low rate. Furthermore, the second peaks of N_2_O emission were 221.1 μg N_2_O-N·m^-2^·h^-1^, 380.1 μg N_2_O-N·m^-2^·h^-1^, 709.2 μg N_2_O-N·m^-2^·h^-1^ and 521.4 μg N_2_O-N·m^-2^·h^-1^ in the control, M, FN and NM pots respectively, which were observed 10 d before the rice harvest (during the period of natural fall of soil surface water).

Applying CMV as GM (M and NM treatments) mitigated the N_2_O emission throughout the rice plants growing periods, especially during the late growing stage which experienced a process of alternation of wetting and drying (Fig 2). Hence it can be argued that incorporation of CMV as GM would be a feasible practice which has a potential to mitigate N_2_O emission from rice-based cropping ecosystem.

Adding nitrogen nutrient (either inorganic or organic N) to paddy soil significantly increased the cumulative emission amounts of N_2_O by 1.13–4.21 folds over the control pots (Table 1). Moreover, incorporating CMV as GM remarkably decreased the cumulative emission amounts and emission factor of N_2_O under the same amount of N nutrient (Table 1). Compared with the FN pots, the cumulative amounts and emission factor of N_2_O were significantly decreased by 59.1% and 73.4% in the M pots, and by 45.2% and 55.9% in the NM pots, respectively.

**Table 1:** The effects of CMV as GM on cumulative amount and emission factor of N_2_O in the monocropped rice system during the growing period of rice plants.

Treatments	Cumulative amounts of N_2_O (kg N_2_O -N ha^-1^)	N_2_O emission factor (%)
Control	0.62±0.04 d	——
M	1.32±0.33 c	0.195±0.01 c
FN	3.23±0.21 a	0.732±0.15 a
NM	1.77±0.11 b	0.323±0.03 b

Data are means ± standard error (n = 3). Different letters within the same column indicate significant differences among treatments at *P* < 0.05.

### The rice grain yield, global warming potential (GWP) and greenhouse gas intensity (GHGI) of N_2_O

Obviously, inputting external nitrogen nutrient into paddy soil notably increased the rice grain yield, GWP and GHGI over a 100-year time horizon by 63.1%-87.5%, 1.12–4.21 folds and 29.6%-185.2% versus the control pots respectively (Table 2).

**Table 2:** Effect of CMV as GM on rice grain yield, GWP and GHGI of N_2_O from monocropped rice field.

Treatments	Rice grain yield (Mg ha^-1^)	GWP (kg CO_2_-eq ha^-1^)	GHGI (kg CO_2_-eq kg^-1^ grain)
Control	3.44±0.22 c	184.9±72.6 d	0.054±0.023 d
M	5.61±0.21 b	392.1±53.3 c	0.070±0.005 c
FN	6.26±0.17 a	963.1±45.9 a	0.154±0.031 a
NM	6.45±0.15 a	527.9±91.8 b	0.082±0.011 b

Data are means ± standard error (n = 3). Different letters within the same column indicate significant differences among treatments at *P* < 0.05.

In addition, under an equal amount of external N nutrient, integrating FN with CMV as GM was able to obtain higher rice grain yield by 3.04% over the FN pots (*P*>0.05), however, applying CMV as GM alone (M) dramatically decreased the rice grain yield by 10.4% when compared with the yield of FN treatment (*P*<0.05) (Table 2).

Moreover, incorporating CMV as GM into paddy soil significantly decreased the GWP and GHGI of N_2_O from rice pots under the same amount of N nutrient (Table 2). Comparing with the FN treatment, the M and NM treatments significantly decreased the GWP by 59.3% and 45.2%, and the GHGI by 54.5% and 46.8%, respectively (*P*<0.05).

### Inorganic N contents in paddy soil

**Fig 3:**
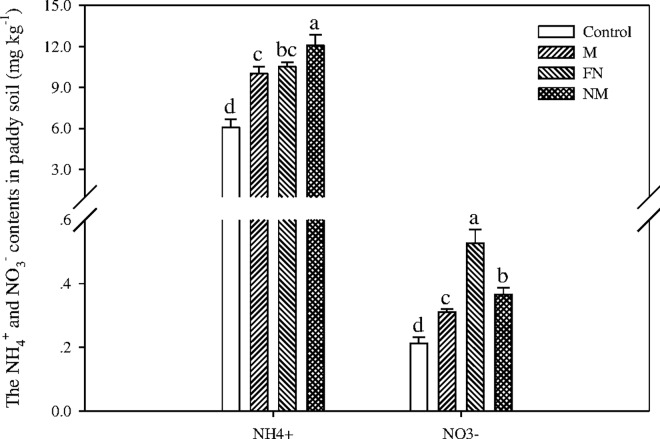
Effects of CMV as GM on the NO_3_^-^ and NH_4_^+^ contents in paddy soil at maturity stage of rice plants. Bars indicate standard error (n = 3). Different letters indicate significantly different means at *P* < 0.05.

The pots in which FN was integrated with CMV as GM increased the NH_4_^+^ content by 14.9% in the plow layer of paddy soil when compared with the FN pots, while the M pots decreased the NH_4_^+^ content by 6.47% versus FN pots under an equal amount of external N nutrient (Fig 3). In addition, incorporating CMV as GM (treatments of M and NM) dramatically decreased the NO_3_^-^ content in paddy soil by 30.7% and 41.1% in comparison to FN pots (Fig 3).

### Populations of nitrifying and denitrifying microbes in paddy soil

**Fig 4:**
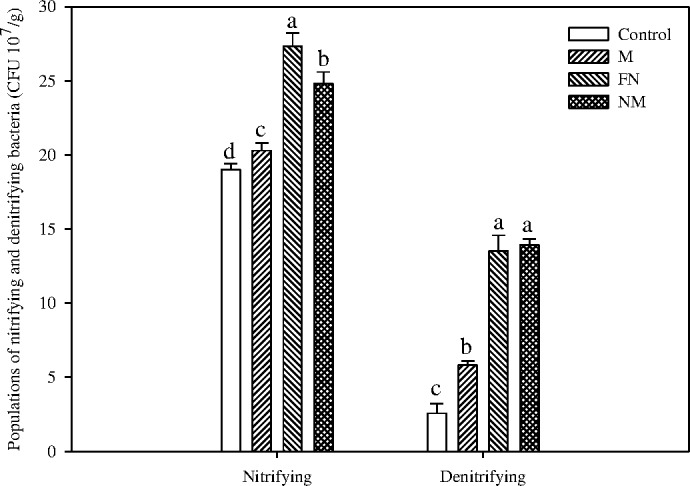
Effect of CMV as GM on the populations of nitrifying and denitrifying bacteria in paddy soil at maturity stage of rice plants. Bars indicate standard error (n = 3). Different letters indicate significantly different means at *P* < 0.05.

In an intelligible manner, applying external N nutrients (either from FN or CMV) significantly increased the populations of nitrifying and denitrifying bacteria by 30.5%-59.4% and 4.26–5.15 fold over the control pots respectively (Fig 4).

Furthermore, under the same amount of external N nutrient, the treatment of incorporating CMV as GM alone (M) dramatically decreased the populations of nitrifying and denitrifying bacteria by 25.7% and 57.0% respectively. Similarly, the NM treatment notably decreased the population of nitrifying bacteria by 9.16% versus the treatment of FN alone (Fig 4).

### Activities of nitrate reductase (*NaR*) and nitrite reductase (*NiR*) in paddy soil

**Fig 5:**
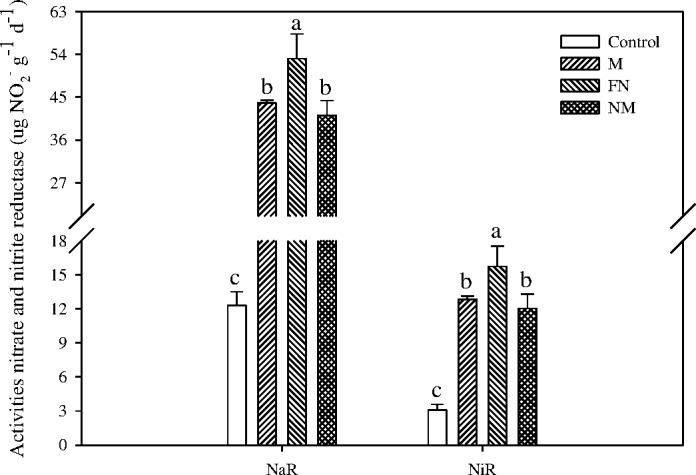
Effects of CMV as GM on the activities of nitrate and nitrite reductase in paddy soil at maturity stage of rice plants. Bars indicate standard error (n = 3). Different letters indicate significantly different means at *P* < 0.05.

Understandably, applying external N nutrient to paddy soil remarkably increased the activities of *NaR* and *NiR* in plow soil layer by 2.34–3.31 folds and 2.91–4.11 folds over the control pots respectively (Fig 5).

Furthermore, incorporating CMV as GM significantly decreased the activities of *NaR* and *NiR* in the plow layer of paddy soil by 17.6%-22.5% and 18.4%-23.5%, respectively, when compared with the treatment of applying fertilizer nitrogen alone (FN) (Fig 5).

## Discussion

Applying external nitrogen nutrient significantly promoted N_2_O emission from paddy soil (Fig 2). Nitrogen fertilizers (e.g. urea, etc.) are decomposed by soil enzymes (e.g. urease, etc.) and thus quickly increased the available inorganic N contents in the plow layer of paddy soil [26]. Furthermore, it has been reported that the exogenous N is more efficiently converted into N_2_O than the soil indigenous N [22].

The N_2_O fluxes peaked on 9^th^ day after basal fertilization and 10^th^ day before rice harvest respectively in all treatments (Fig 2). Though ample N_2_O is emitted from paddy soil in both the waterlogged and AMD (alternate wetting and drying) conditions, soil water content directly affects production and consumption of N_2_O, through its influence on micro-organisms and root activity, especially during the AMD process [48].

In this study, the leguminous CMV plants, which can fix atmospheric N [49], were seeded at a rate of 30 kg ha^-1^ on the non-tilled reddish paddy soil with residual moisture after rice harvest in the last-ten days of September, 2012. The growing period mainly consisted of winter and spring seasons when fields are normally fallow [50]. No fertilizers were applied to CMV plants during their growing periods and water was manually sprayed each week to keep suitable soil moisture. Our findings showed that the incorporation of CMV as GM (M and NM) into paddy soil notably inhibited the N_2_O emission from monocropped rice system over the treatment of FN alone (Fig 2 and Table 1). The flux of N_2_O emission between the interface of soil and the atmosphere largely depends on O_2_ availability and N substrate availability as NO_3_^-^ and NH_4_^+^ [16]. Firstly, the decrease in N_2_O emission might be resulted from the demands of substrate available C and N during the decomposition of cover crop residues for self-informing the cells of microbes in paddy soil [51]. Secondly, the decomposition of cover crop residues by soil microbes produced lots of active reducing substances (e.g. phenol, etc.) and decreased the oxidation-reduction potential (ORP), which might further deoxidize the N_2_O to NO or N_2_ by denitrification process [52–53], and then decreased the N_2_O emission between the interface of soil and the atmosphere. Thirdly, allelochemicals (e.g. benzoic acid and *p*-tert-benzoic acid, etc.) produced from the decomposition of cover crop residues could inhibit soil N_2_O production [54], since it leads to a net immobilization of soil N during the early stage of decomposition of legume crop residues which has a low C/N ratio [55]. As a result, the residual CMV presumably led to nitrogen immobilization in the paddy soil, decreasing N substrate for nitrification and denitrification and in turn N_2_O/N_2_ ratio and N_2_O emission from the soil [56]. In addition, both of *NaR* and *NiR* are key enzymes in the process of nitrification and denitrification, especially the *NiR* is vital to the process of denitrification [57]. Microbial denitrification is the main source of N_2_O evolved from soil [58]. Our results showed that integrating CMV as GM with FN decreased the activities of *NaR* and *NiR* (Fig 5), which presumably decreased the amounts of electron acceptor (NO_2_^-^) of denitrification and in turn the N_2_O fluxes in paddy soil.

Although, it is worth-mentioning that raising CMV requires time, land, labor, irrigational water and several other types of related resources. Thus, while considering the effectiveness of using CMV as GM, all these factors should be kept in mind especially when the focus is on to determine its economical importance in terms of cost benefit ratio. That is being said, the intensively fertilized and irrigated systems usually present high levels of mineral N, which tends to be lost through denitrification during the fallow periods after the main crop harvest [59]. Hence, replacing bare fallow with catch crops (e.g. CMV) has been considered as reasonable technique to decrease the losses of post-harvest surplus inorganic N [60]. Due to the N and water demand of catch crops, changes in soil moisture, N pool may occur in the catch crop growing period, and thus influencing the processes of N_2_O emission. Though to date, there was a few of information concerning the effect of catch crop on N_2_O emission [60], and also more further long-term filed experiments should be carried out to evaluate the impact of planting CMV as catch crop after the rice harvest on greenhouse gases (e.g. N_2_O) emission from the reddish paddy soil in south China.

Previously it has been shown that fertilizer N application rates influenced N_2_O emission from agricultural soils [61]. For instance, if the N fertilization rate was sub- or equal to those required for maximum yields, N_2_O emission increased linearly with increasing N fertilizer rates [62], while it became more variable and increased exponentially with the increasing N fertilizer rates under condition of exceeding plant requirement [61]. Furthermore, the abundance of soil NO_3_^-^-N was one of the most important factors affecting N_2_O emissions from fertilized fields [63]. According to Xie et al. (2016) [30], the NO_3_^-^-N content decreased when N fertilizer was partially substituted by CMV in paddy soil. They further reported that this substitution of commercial N by CMV at higher rates (e.g. substituting 60%-80% FN by GM) resulted in a yield penalty for double rice systems. Hence, it is important to go for best management practices for fertilizer N as it plays a vital role in ensuring higher rice grain yield and minimizing residual soil NO_3_^-^-N, which will reduce the risk of increased N_2_O emissions from agricultural soils [61]. As a result, it seems logical to assess the N_2_O emission in the light of the amount of N utilized for rice grain yield formation, i.e. yield-based N_2_O emissions [58].

Compared to the FN pots, both the M and NM pots dramatically decreased the GWP and GHGI of N_2_O from paddy soil (Table 2). The content of soil organic matter reduced along with the decrease of soil pH which was a result of applying synthesized nitrogenous fertilizers [64–65]. However, applying organic manures (e.g. GM, etc.) not only efficiently increased the soil pH and SOC content, but also decreased the depletion rate, which promoted the accumulation of SOC in paddy soil [66–68]. According to West and Six (2007) [69], the cumulative SOC resulted in a decrease of GWP, but the extent is narrowed as the content gradually reaching the saturation of SOC in soil. Hence, it is necessary for establishing long term field experiment to in-situ monitor the effect of incorporating GM on GWP from monocropped rice ecosystems. In addition, it is clear that a decrease in the GWP caused a decline of GHGI. Moreover, the crop yield also is an important factor that influences the GHGI in agro-ecosystems [70] and the net effect of higher yields have offset emission by as much as 590 Gt CO_2_-eq since 1961 [71]. Similarly, under the condition of the equal amount of N, integrating CMV as GM with FN increased the rice grain yield which should result in a decrease of the GHGI of N_2_O from the monocropped rice system (Table 2).

Yet, we just evaluated the effect of GM on the GWP and GHGI of N_2_O during the rice plants growing periods with a pot-culture experiment, it is imperative to assess the influence of GM on the GWP and GHGI of CO_2_ and CH_4_ under field conditions. Moreover, in order to comprehensively examine the sustainability of GM in the paddy ecosystem, it is also important to monitor the emissions of N_2_O, CO_2_ and CH_4_ during growing period of CMV plant.

Moreover, incorporating FN with CMV dramatically decreased the NO_3_^-^ content and the process of nitrification, whereas it increased the NH_4_^+^ content in paddy soil (Figs 3 and 4). This can be a result of increasing SOC content from CMV residue addition, which increased NH_4_^+^ simply due to mineralization of crop residue. In addition, incorporating cover crop (e.g. ryegrass, etc.) residues as GM remarkably increased the SOC in paddy soil [68], which presumably promoted the process of dissimilatory nitrate reduction to ammonium (DNRA) and significantly increased the NH_4_^+^ content in soil. However, the high NH_4_^+^ concentration inhibited the growth and population of nitrifying bacteria as well as the nitrification rate, which in turn decreased the NO_3_^-^ content in top soil layer. Furthermore, as mentioned above, incorporating cover crop residues decreased the ORP in soil [52–53], which presumably depressed the growth of nitrifying bacteria and the nitrification process. In addition, incorporating CMV to paddy soil depressed the activities of *NaR* and *NiR* (Fig 5), it was implying that the process of reducing NO_3_^-^ to NO_2_^-^ might also decrease the population of nitrifying bacteria in top paddy soil.

## Conclusion

Application of CMV as GM alone or its combination with FN to paddy soil not only mitigated N_2_O emission when compared with FN treatment but also minimized the GWP and GHGI of N_2_O during paddy growth period. Incorporating CMV as GM into paddy soil also depicted that reduced population of nitrifying bacteria and activities of *NaR* and *NiR* which led to decreasing the NO_3_^-^ content in top paddy soil at the maturity stage of rice. However, application of CMV as GM alone significantly decreased the rice grain yield and soil NH_4_^+^ content which were increased in the treatment of integrating FN with CMV, over the treatment of FN alone.

These results implied that integrating FN with CMV as GM is more beneficial for obtaining more rice grain yield and enhancing N-retaining capacity of paddy soil, while mitigating N_2_O emission as well as minimizing the environmental risks related to NO_3_^-^ leaching or runoff from the flooded monocropped rice ecosystem. Nevertheless, all the results should be further monitored by long term field experiments and under different soil type as well as eco-environmental conditions. Moreover, the unrevealing potential impacts of GM on the annual GHGs (CH_4_, CO_2_ and N_2_O) emission as well as the net-GWPs and GHGI are worth further investigation to determine sustainable cycles of GM in the monocropped rice ecosystem.
